# Preparation of Lipid Cubic Liquid Crystalline Nanoparticles of Sinomenine Based on Molecular Dynamics Simulations and Investigation of the Efficacy Against Rheumatoid Arthritis

**DOI:** 10.3390/ijms262110773

**Published:** 2025-11-05

**Authors:** Jiaoyue Zhu, Jingying Li, Yunlu Zou, Xuehui Ding, Jixin Li, Jiahui Xu, Yinghao Xiao, Ye Qiu, Wei Xu

**Affiliations:** School of Pharmacy, Changchun University of Chinese Medicine, Changchun 130117, China; 15834878190@163.com (J.Z.); 18043910903@163.com (J.L.); 17390991885@163.com (Y.Z.); 15837573625@163.com (X.D.); 18343412752@163.com (J.L.); 15590142627@163.com (J.X.); 13517376162@163.com (Y.X.)

**Keywords:** molecular dynamics simulations, Sinomenine, lipid cubic liquid crystalline nanoparticles, in situ single-pass intestinal perfusion study

## Abstract

Sinomenine (SIN) is a promising candidate for the treatment of rheumatoid arthritis (RA). Although it possesses the advantage of being non-addictive, its poor aqueous solubility and low oral bioavailability have limited its clinical application. To address these issues, SIN was encapsulated into lipid cubic liquid crystal nanoparticles (LCNPs) and systematically characterized. Molecular dynamics (MD) simulations were first employed to screen suitable excipients for formulation development. Combined with single-factor optimization and Box–Behnken response surface design, the optimal composition and preparation process were determined. The resulting SIN-LCNPs exhibited a particle size of 149.7 ± 0.9 nm, a polydispersity index (PDI) of 0.223 ± 0.01, a zeta potential of −18.9 mV, and an encapsulation efficiency (EE%) of 92.2%. Spectroscopic analyses confirmed successful incorporation of SIN into the lipid matrix. Pharmacodynamic studies revealed that SIN-LCNPs enhanced targeted drug delivery to inflamed joints, significantly alleviating inflammation and suppressing disease progression in rats. In vivo single-pass intestinal perfusion (SPIP) experiments further demonstrated that SIN was primarily absorbed through the small intestine and that the LCNP carrier effectively improved its intestinal permeability. Collectively, this study provides a novel strategy and theoretical foundation for developing efficient formulations of poorly water-soluble drugs, highlighting the potential clinical application of SIN-LCNPs in RA therapy.

## 1. Introduction

Rheumatoid arthritis (RA) is a chronic systemic autoimmune disorder characterized by symmetrical swelling and pain in joints such as the hands, wrists, and feet [1]. It is marked by synovial inflammation and joint cell infiltration, ultimately leading to joint destruction and bone erosion. The global prevalence of RA ranges from 0.5% to 1.0%. Non-steroidal anti-inflammatory drugs (NSAIDs) and glucocorticoids are commonly prescribed for RA management [2], but their application is limited by low response rates, adverse effects, and safety concerns. Sinomenine (SIN) is an isoquinoline alkaloid isolated from the roots and stems of Sinomenium acutum (Thunb.) Rehd. et Wils., a climbing shrub of the family Menispermaceae. SIN exhibits potent anti-inflammatory, analgesic, and immunosuppressive effects [3], making it a promising therapeutic candidate for RA without addictive potential. However, its poor water solubility and low bioavailability have limited its clinical application [4]. In recent years, nanomaterials have been widely applied in drug delivery systems, and the integration of bioactive compounds from traditional Chinese medicine with nanotechnology has attracted growing interest for disease treatment and diagnosis.

Lyotropic Liquid Crystals (LLCSs) are formed by the self-assembly of amphiphilic surfactant molecules in water or other polar solvents [5]. These systems include lamellar (Lα), hexagonal (H_2_), and cubic (V) phases. Liposomes (LIPOs) are derived from the lamellar phase, whereas dispersions of the bicontinuous cubic phase in water form lipid cubic liquid crystalline nanoparticles (LCNPs) [6]. Unlike LIPOs, which encapsulate drugs within a simple lipid bilayer, LCNPs undergo rearrangement into cubic or hexagonal phases, providing both hydrophilic and lipophilic domains. This unique structure allows LCNPs to encapsulate drugs with different polarities, improve water solubility, and enhance bioavailability [7]. In addition, LCNPs exhibit nanoscale properties and can be chemically or biologically modified for targeted drug delivery. Their bioadhesive nature enables binding to the intestinal mucosa, prolonging drug residence time. Encapsulation of biomolecules (e.g., proteins, amino acids) within LCNPs protects them from enzymatic degradation in the gastrointestinal tract, thereby reducing drug loss and improving systemic absorption [8]. Previous studies have demonstrated the potential of LCNPs. For instance, an orally administered curcumin-loaded LCNP formulation enhanced relative bioavailability by nearly 15-fold compared with free curcumin [9], while a vitamin B_12_-loaded LCNP system improved targeting and controlled release, which showed particular affinity for bone marrow and neural cells [10].

In recent years, molecular dynamics (MD) simulations have been increasingly applied in pharmaceutical formulation development. As a technique that mimics laboratory conditions at the molecular level, MD enables accurate characterization of microscopic dynamic changes within a system and provides mechanistic insights into experimental phenomena, thereby enhancing research efficiency, cost-effectiveness, and predictive accuracy [11]. Before formulation development, MD plays a crucial role in screening drug carriers and identifying excipients with high drug loading capacity, markedly improving efficiency through reliable predictive insights. Importantly, MD complements conventional experimental methods by providing mutual validation, and their integration facilitates iterative refinement of formulations through combined macroscopic and microscopic investigations [12].

This study represents the first attempt to incorporate SIN into LCNPs to address its poor water solubility, low bioavailability, and limited targeting of RA lesions. Unlike conventional carriers that alleviate individual physicochemical issues, LCNPs simultaneously overcome these challenges. Their nanoscale size enables passive targeting through the enhanced permeability and retention (EPR) effect, facilitating drug accumulation in inflamed joints. The single-pass intestinal perfusion (SPIP) experiment further confirmed the enhanced intestinal absorption capacity, supporting its potential for oral formulation development. Distinct from generalized compatibility screening in MD-based formulation studies, this work evaluated the molecular conformational compatibility and encapsulation stability between SIN and LCNP components. Integration of MD simulations with experimental characterization established a closed-loop framework that provides molecular-level guidance for formulation design and minimizes empirical trial and error. Beyond SIN formulation optimization, this research proposes a comprehensive R&D framework integrating MD-based excipient screening, response surface optimization, in vivo pharmacodynamic evaluation, and intestinal absorption validation. This systematic approach not only ensures safety and targeting efficiency for long-term RA therapy but also offers a reproducible and practically implementable strategy for the rational design of nanocarriers for other hydrophobic therapeutic agents.

## 2. Results

### 2.1. Excipient Screening

The solubility parameters (δ) of the drug and various excipients are presented in Table 1. As shown, SIN shows a relatively small solubility difference with glycerol monolaurate (GMO) and phytolactone (PT) and the smallest solubility difference with poloxamer 407 (P407), satisfying the ∆δ < 7.5 compatibility requirement. Simulation results show that SIN has superior compatibility with GMO, PT, and P407 compared to the other five excipients. Thus, P407 is selected as the stabilizer for preparing SIN-LCNPs, with GMO and PT selected as the lipids.

The experimental validation results for drug–excipient compatibility are summarized in Table 2. As shown, when GMO was used as the lipid material, the cubic liquid crystal nanoparticles had a particle size of 162.8 nm and an encapsulation efficiency (EE%) of 86.6%. This formulation exhibited both the smallest particle size and the highest encapsulation efficiency. Thus, GMO was selected as the lipid material for preparing SIN-LCNPs, in agreement with the MD simulations results.

### 2.2. Optimization of SIN-LCNP Formulation and Process

**Figure 1:**
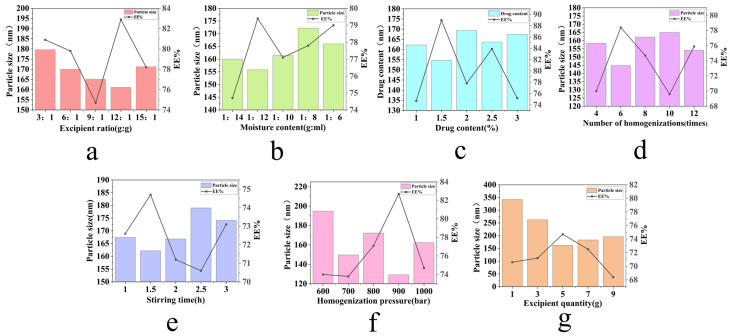
Single-factor investigation of SIN-LCNP formulation and process. (**a**) Effect of different excipient ratios on particle size and EE%. (**b**) Effect of different moisture contents on particle size and EE%. (**c**) Effect of different drug content on particle size and EE%. (**d**) Effect of different numbers of homogenizations on particle size and EE%. (**e**) Effect of stirring time on particle size and EE%. (**f**) Effect of homogenization pressure on particle size and EE%. (**g**) Effect of excipient quantity on particle size and EE%. Results are expressed as mean ± SD (*n* = 3).

Formulation design and process parameters directly influence the drug loading efficiency and release performance of cubic liquid crystal drug delivery systems. Therefore, a systematic screening of formulation components and preparation processes was conducted to optimize the formulation outcomes. Initial single-factor investigations (Figure 1) showed that the lowest particle size and highest EE% of SIN-LCNPs were achieved with 5 g excipient, 1.5 h mixing, 900 bar homogenization pressure, and 6 homogenization cycles. As the excipient ratio was adjusted, EE% increased initially, peaked, and then declined. Particle size first decreased, then increased. At a 12:1 excipient ratio, EE% reached a maximum of 82.9%, while particle size decreased to 161.2 nm. Therefore, the subsequent optimization focused on three ratios: 9:1, 12:1, and 15:1. Regarding moisture content, EE% first increased and then decreased, while the particle size showed a similar ‘decrease-then-increase’ trend. At a 1:12 moisture content ratio, EE% reached a maximum of 79.4%, with particle size decreasing to 155.8 nm. Based on these results, subsequent optimization focused on three moisture content gradients: 1:14, 1:12, and 1:10. During drug loading adjustment, EE% increased to a peak before declining, while particle size first decreased, then increased. At 1.5% drug loading, EE% peaked at 89.0%, and particle size decreased to 154.7 nm. Thus, subsequent optimization focused on three drug content levels: 1%, 1.5%, and 2.0% for further investigation.

**Figure 2:**
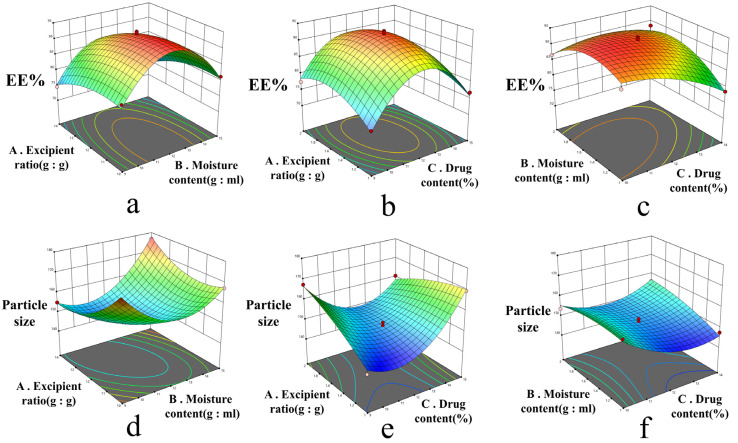
Response surface plots for the interaction effects of various factors in the optimization of the SIN-LCNP formulation using the Box–Behnken design and effect surface method. (**a**) Effect of excipient ratio and moisture content on EE%; (**b**) Effect of excipient ratio and drug content on EE%. (**c**) Effect of moisture content and drug content on EE%. (**d**) Effect of excipient ratio and moisture content on particle size. (**e**) Effect of excipient ratio and drug content on particle size. (**f**) Effect of moisture content and drug content on particle size.

This study employed Design-Expert 13.0 software for response surface modeling and analysis of variance (ANOVA). The test design and results are shown in Table 3. For indicator Y_1,_ ANOVA results are shown in Table 4. The fitted regression equation was Y_1_ = 92.62 − 0.3250A − 3.05B + 1.05C − 0.1500AB − 1.15AC + 3.05BC − 12.31A^2^ − 2.56B^2^ − 4.41C^2^ (R^2^ = 0.9764, R^2^adj = 0.9461). ANOVA indicated that the model was highly significant (*p* < 0.0001) with an insignificant lack of fit (*p* = 0.3319 > 0.05), demonstrating good overall fitness. Factor B and its interaction term BC had significant effects on Y_1_ (*p* < 0.05), whereas the quadratic terms A^2^ and C^2^ showed highly significant effects (*p* < 0.01). The quadratic term B^2^ also exhibited a significant influence (*p* < 0.05). For indicator Y_2_, ANOVA results are shown in Table 5. The fitted equation was Y_2_ = 149.66 + 3.76A − 2.71B + 2.07C + 7.82AB − 7.80AC + 3.45BC + 12.96A^2^ + 6.36B^2^ − 2.02C^2^ (R^2^ = 0.9844, R^2^adj = 0.9643). ANOVA revealed a highly significant model fit (*p* < 0.0001) with an insignificant lack of fit (*p* = 0.1448 > 0.05), confirming a good model adequacy. Factors A and B exhibited highly significant effects on Y_2_ (*p* < 0.01), while factor C had a significant effect (*p* < 0.05). The interaction terms AB, AC, and BC were also highly significant (*p* < 0.01). Furthermore, the quadratic terms A^2^ and B^2^ had highly significant impacts on Y_2_ (*p* < 0.01). Response surface plots (Figure 2) were generated based on the regression equation, following ANOVA results for the regression model. Steeper response surface curves indicate more pronounced factor effects [13]. The Box–Behnken design-effect surface method was used to analyze the effects of excipient ratio, moisture content, and drug content on EE% and particle size, yielding the optimal formulation: excipient ratio 11.774:1, moisture content 11.890:1, and drug content 1.435%. For convenience and weighing accuracy, the final formulation was determined to be excipient ratio 11.8:1, moisture content 11.9:1, and drug content 1.4%.

### 2.3. Characterization of SIN-LCNPs

#### 2.3.1. Appearance, Particle Size, Polydispersity Index (PDI), and Zeta Potential

**Figure 3:**
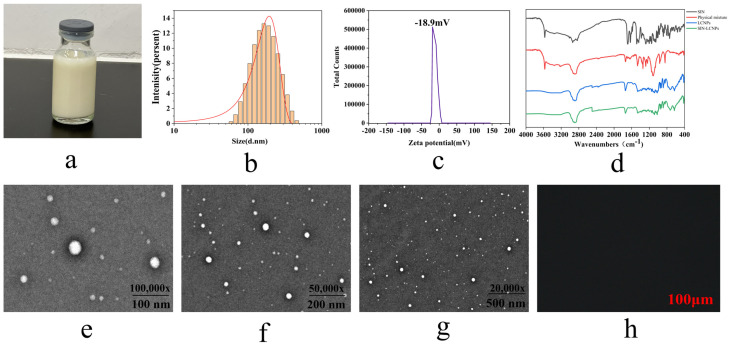
Characterization of SIN-LCNPs. (**a**) Photograph of SIN-LCNPs. (**b**) Particle size distribution of SIN-LCNPs. (**c**) Potentiogram of SIN-LCNPs. (**d**) FT-IR spectra of SIN, physical mixture, LCNPs, and SIN-LCNPs. (**e**–**g**) TEM images of SIN-LCNPs. (**h**) PLM detection image of SIN-LCNPs.

As shown in Figure 3a, the SIN-LCNPs prepared in this study presented as a pale yellow homogeneous emulsion. The average particle size was 149.7 nm (Figure 3b), with a PDI of 0.223 ± 0.01 and a zeta potential of −18.9 mV (Figure 3c). These results indicate that the nanoparticles possessed a small particle size, a narrow size distribution, and a zeta potential absolute value below 30 mV, suggesting good stability of the prepared SIN-LCNPs.

#### 2.3.2. Fourier Transform Infrared Spectroscopy (FT-IR) Testing

As shown in Figure 3d, FT-IR spectroscopy provides clear evidence of the successful encapsulation of SIN within the LCNP matrix, as indicated by the characteristic peak shifts and disappearance of specific functional group signals. The spectra show that free SIN exhibits a C =O stretching vibration at 1688.3 cm^−1^ and an –OH stretching vibration at 3573.2 cm^−1^. In SIN-LCNPs, both of these peaks disappear, which can be attributed to hydrogen bonding and steric hindrance between the C=O and –OH groups of SIN and the ester groups or alkyl chains of GMO, resulting in the masking or significant shifting of these vibrational modes. The persistence of GMO’s characteristic peaks—methine and methylene stretching vibrations (2800–3000 cm^−1^) and ester carbonyl absorption (1730–1750 cm^−1^)—with slightly altered shapes further supports that SIN is not simply physically mixed with GMO, as would be evidenced by the retention of SIN’s characteristic peaks. Instead, SIN is molecularly encapsulated within the lipid matrix of LCNPs through intermolecular interactions, leading to the modification or disappearance of its signature vibrations. These findings conclusively confirm the encapsulated state of SIN within the LCNPs.

#### 2.3.3. Transmission Electron Microscopy (TEM) Morphological Examination

As shown in Figure 3e–g, the imaging results indicate that SIN-LCNPs possess a nearly spherical three-dimensional morphology with no evidence of aggregation or adhesion between particles. This stability can be attributed to electrostatic repulsion arising from abundant positive charges on the nanoparticle surfaces, which ensures stable dispersion [14]. During sample preparation using the phosphotungstic acid negative staining method, the concentration and amount of staining solution markedly affect particle contrast. Inappropriate droplet application or drying may result in localized particle aggregation. To minimize such artifacts, sample preparation conditions in this study were carefully controlled by diluting samples to suitable concentrations and ensuring uniform drying. These precautions effectively eliminated interference and ensured that the morphology observed in TEM images accurately reflected the true dispersed state of the nanoparticles.

#### 2.3.4. Polarized Light Microscopy (PLM) Analysis

As shown in Figure 3h, SIN-LCNPs exhibited a dark-field appearance under PLM without birefringence, which is consistent with the optical isotropy of cubic liquid crystals. These observations suggest that the nanoparticles possessed a cubic liquid crystal phase structure.

### 2.4. Pharmacodynamic Study of SIN-LCNPs in AA Rats

**Figure 4:**
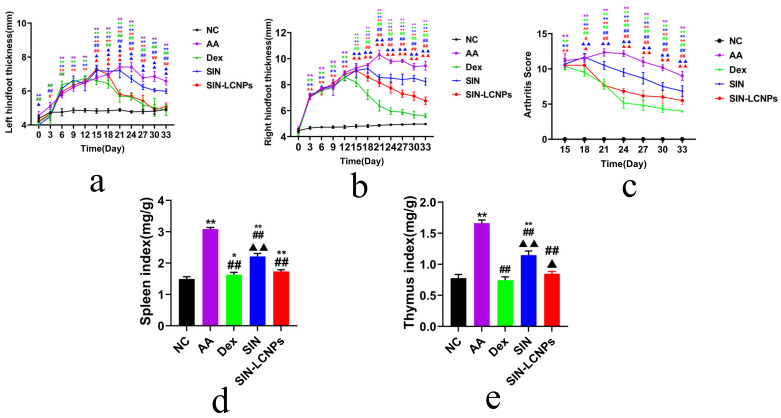
Pharmacodynamic study of SIN-LCNPs in AA rats. (**a**) Left paw thickness in each group. (**b**) Right paw thickness in each group. (**c**) Arthritis index scores in each group. (**d**) Spleen indices in each group, (**e**) Thymus indices in each group. (*n* = 6, * *p* < 0.05, ** *p* < 0.01, compared with the normal group; ^#^ *p* < 0.05, ^##^ *p* < 0.01, comparing the Dex group, SIN group and SIN-LCNP group with the AA group; ^▲^ *p* < 0.05, ^▲▲^ *p* < 0.01, comparing the SIN and SIN-LCNP groups with the Dex group).

As shown in Figure 4a,b, both SIN and SIN-LCNP groups exhibited gradual reductions in paw swelling over time. The SIN group showed only slight and non-significant decreases in joint swelling between days 21 and 24, whereas the SIN-LCNP group achieved significant reductions from day 15 onwards, demonstrating notably enhanced efficacy. Arthritis index scores (Figure 4c) progressively decreased in both groups with continued dosing, with greater reductions observed in the SIN-LCNP group. These findings suggest that encapsulation within liquid crystal carriers facilitates more efficient drug delivery to inflammatory sites, thereby improving therapeutic efficacy. Rat spleen and thymus indices are presented in Figure 4d,e.

**Figure 5:**
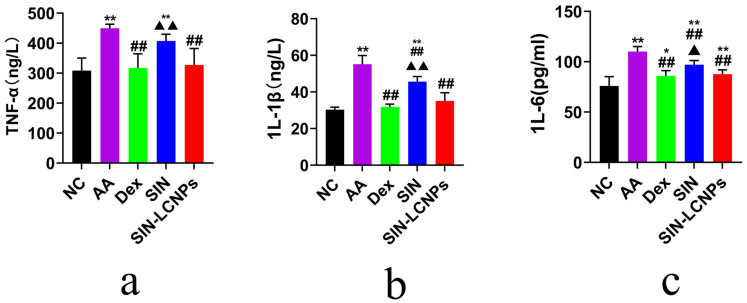
Pharmacodynamic study of SIN-LCNPs in AA rats: serum inflammatory cytokine levels at 33 days post-treatment across different groups. (**a**) TNF-α; (**b**) IL-1β; (**c**) IL-6; *n* = 6, * *p* < 0.05, ** *p* < 0.01, comparing different groups with the normal group; ^##^ *p* < 0.01, comparing the Dex group, SIN group, and SIN-LCNP group with the AA group; ^▲^ *p* < 0.05, ^▲▲^ *p* < 0.01, comparing the SIN group and SIN-LCNP group with the Dex group).

**Figure 6:**
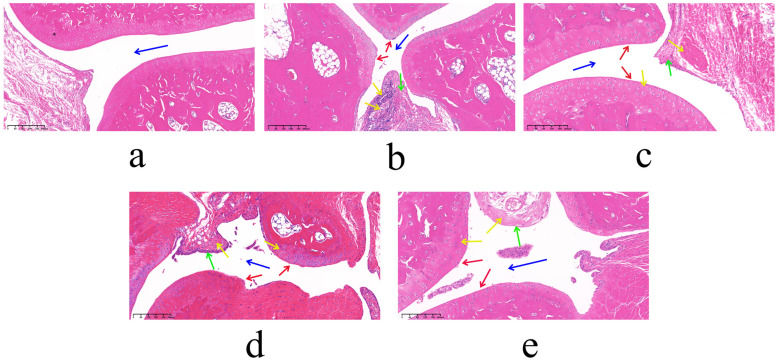
Pharmacodynamic study of SIN-LCNPs in AA rats: HE sections of joints from each group. (**a**) NC group; (**b**) AA group; (**c**) Dex group; (**d**) SIN group; (**e**) SIN-LCNP group. (*n* = 6, blue arrows indicate joint cavities; red arrows indicate cartilage surfaces; green arrows indicate synovial cells; yellow arrows indicate the degree of inflammatory cell infiltration in the tissue).

Compared with the normal control (NC) group, AA rats exhibited markedly enlarged spleens and thymuses with darkened coloration and significantly elevated indices. In contrast, the SIN-LCNP group showed significantly reduced spleen and thymus indices relative to the AA group, indicating attenuation of systemic inflammatory responses and suppression of arthritis progression. Serum inflammatory cytokine measurements (Figure 5) revealed lower concentrations of tumor necrosis factor-alpha (TNF-α), interleukin-1 beta (IL-1β), and interleukin-6 (IL-6) in the SIN-LCNP group compared with the SIN group, indicating significant suppression of pro-inflammatory cytokine secretion and alleviation of RA severity. Histological examination (HE) (Figure 6) showed normal joint tissue structure in the NC group, characterized by smooth cartilage surfaces and evenly distributed chondrocytes. In contrast, the AA group displayed severe pathological changes, including synovial hyperplasia, cartilage erosion, and extensive inflammatory cell infiltration. All treatment groups improved these pathological features to varying degrees, with the dexamethasone (Dex) and SIN-LCNP groups exhibiting the most pronounced amelioration of synovial hyperplasia, bone destruction, and inflammatory infiltration, with significant differences compared with the SIN group.

Overall, SIN-LCNPs exhibited significantly improved efficacy compared with free SIN, as evidenced by greater reductions in joint swelling and arthritis scores, suppression of pro-inflammatory cytokine secretion, and modulation of immune organ indices. Collectively, these findings indicate that SIN-LCNPs provide enhanced therapeutic effects in a rat model of RA.

### 2.5. SPIP Study of SIN-LCNPs

**Figure 7:**
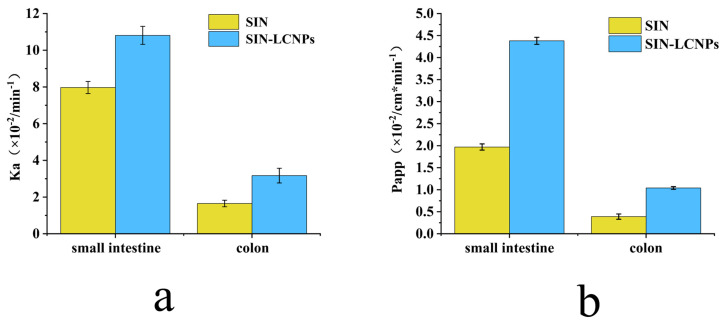
Comparison of in situ absorption of SIN solution and SIN-LCNPs in rat small intestine and colon. (**a**) Ka; (**b**) Papp. Results are expressed as mean ± SD (*n* = 3).

As shown in Figure 7, both SIN and SIN-LCNPs exhibited significantly higher absorption rate constant (Ka) and apparent permeability coefficient (Papp) values in the small intestine than in the colon (*p* < 0.05 or *p* < 0.01), indicating more efficient drug absorption in the small intestinal segment. SIN, a Biopharmaceutics Classification System (BCS) Class II drug, exhibits limited oral absorption due to poor aqueous solubility (0.8–1.2 mg·mL^−1^) and low intestinal permeability, with a Papp of (1.97 ± 0.07) × 10^−4^ cm·min^−1^. SPIP studies revealed that SIN-LCNPs markedly enhanced absorption efficiency, showing 1.36- and 1.92-fold increases in small-intestinal Ka and Papp, and 2.22- and 2.67-fold increases in colonic Ka and Papp, respectively. The improved absorption resulted from the synergistic enhancement of solubility and permeability by the LCNP carrier. The lipid-based nanostructure increased SIN dissolution by enlarging surface area and protecting the drug from gastrointestinal degradation. Meanwhile, the lipid matrix mitigated P-glycoprotein (P-gp)-mediated efflux and transiently increased membrane permeability, facilitating cellular uptake and possible lymphatic transport. Consequently, the small-intestinal Papp of SIN-LCNPs reached (4.38 ± 0.08) × 10^−4^ cm·min^−1^, approaching the high-permeability threshold (5 × 10^−4^ cm·min^−1^). These findings suggest that SIN-LCNPs exhibit Class I-like absorption characteristics, providing a promising strategy to improve the oral bioavailability of SIN.

## 3. Summary and Discussion

This study employed MD simulations using the δ as the evaluation metric to screen suitable excipients for the SIN-LCNPs formulation. However, this computational approach has inherent limitations, including assumptions of ideal solution conditions and the neglect of factors such as molecular size variations, differences in crystallinity, and molecular diffusion rates. These simplifications may cause discrepancies between the predicted compatibility and experimentally determined Δδ values. To address these limitations, we conducted follow-up experimental validation using GMO as the lipid component and P407 as the stabilizer. The resulting LCNPs exhibited the smallest particle size and the highest EE%, which were fully consistent with the MD simulation results. This finding confirms the reliability of MD simulations in narrowing the experimental screening scope and providing rational guidance for formulation design. Nevertheless, the obtained δ value for SIN (22.122 (J/cm^3^)^1/2^) showed minor deviations from the literature-reported value (19.957 (J/cm^3^)^1/2^) for similar systems. These discrepancies mainly arise from three factors: (1) the use of different force fields—specifically, the COMPASS II force field adopted in this study offers more accurate descriptions of organic small molecules and their functional group interactions, thereby optimizing parameters for lipid–drug systems; in contrast, the GAFF force field used in previous studies may cause deviations in the cohesive energy density (CED) calculations due to differing charge assignments; (2) variations in conformational and volume calculations between the NVT ensemble (used in this work) and the NPT ensemble (used in the literature); and (3) differences in model construction, as this study employed a solvent-free molecular cell, whereas previous studies used solvent-containing systems, which may weaken the calculated CED values. Importantly, these deviations do not compromise the reliability of the simulation results. Overall, MD simulations provide a solid scientific basis for the preliminary design of experimental formulations. The subsequent experimental work further elucidates the influence of practical factors such as formulation composition and processing parameters on LCNP carriers, building upon the computational predictions. The two approaches complement and validate each other, ultimately enabling the optimization of SIN-LCNP formulations and processes. This integrated strategy highlights the guiding value of computer simulations in the development of nano-drug delivery systems.

In pharmacodynamic studies of SIN-LCNPs, the establishment of an animal model that closely resembles the pathogenesis of human RA is essential for evaluating therapeutic efficacy and elucidating mechanisms of action [15]. In this study, the AA model, induced by injection of CFA into the rat ankle joint, was selected because it exhibits clinical features comparable to human RA and allows rapid model induction [16]. Considering the variation in disease incidence and synchronization within the AA model [17], modeling status was continuously monitored following secondary immunization. All animals successfully developed arthritis within 3–5 days, and drug administration was initiated on day 7 to ensure model reliability. Pharmacodynamic evaluation demonstrated that, compared with free SIN, SIN-LCNPs significantly reduced paw swelling, arthritis scores, and immune organ indices, thereby alleviating clinical symptoms in AA rats. Histopathological analysis further confirmed that SIN-LCNPs effectively mitigated synovial hyperplasia, cartilage destruction, and inflammatory cell infiltration. Although joint damage was improved in all treatment groups, the Dex and SIN-LCNP groups exhibited the most notable protective effects, with significant differences compared with the SIN group. Cytokine analysis provided mechanistic insights into these therapeutic effects. TNF-α, a key autocrine and paracrine regulator, amplifies RA inflammation by promoting the secretion of IL-1, IL-6, and GM-CSF, as well as adhesion molecules such as VCAM-1 and ICAM-1, thereby driving joint and cartilage damage [18,19]. IL-1β, secreted by activated macrophages and synovial cells, induces downstream release of TNF-α and IL-6, further amplifying the inflammatory cascade [20,21]. IL-6 promotes synovial proliferation and osteoclast activation while inhibiting cartilage matrix synthesis, resulting in cartilage erosion and bone resorption [22,23]. In this study, SIN-LCNPs significantly reduced serum TNF-α, IL-1β, and IL-6 levels compared with SIN, suggesting that their therapeutic efficacy may be attributed, at least in part, to modulation of pro-inflammatory cytokine expression and suppression of the inflammatory response.

In terms of anti-inflammatory efficacy, the Dex group showed a trend towards superiority compared with the SIN-LCNP group; however, statistical analysis revealed no significant differences between the two groups (*p* > 0.05). In contrast, significant differences were observed in safety and physiological status. Rats in the Dex group exhibited a markedly lower average body weight (274.3 g) compared with the NC group (425.1 g, *p* < 0.05), and some animals developed mild diarrhea. By comparison, rats in the SIN-LCNP group maintained an average body weight of 404.6 g, with no abnormal physiological responses, and showed no significant difference from the NC group. These findings suggest that, unlike Dex, SIN-LCNPs do not adversely affect growth or general health. The adverse effects observed in the Dex group may be attributed to the well-documented side effects of glucocorticoids, including suppression of the hypothalamic–pituitary–adrenal axis leading to adrenal cortical insufficiency, inhibition of gastrointestinal motility with reduced food intake, and disruption of energy metabolism through altered glucose and insulin regulation [24,25]. Such mechanisms can result in impaired growth and reduced body weight in rats. In summary, while SIN-LCNPs exhibited anti-inflammatory efficacy comparable to Dex, they were associated with no detectable adverse reactions, indicating superior safety and biocompatibility in anti-inflammatory therapy.

Models for studying intestinal absorption of oral drugs primarily include in vivo, in situ, and in vitro approaches [26,27,28]. Among these, the in situ intestinal perfusion model—an in vivo technique—is widely applied to investigate drug absorption and transport mechanisms, as it closely replicates physiological absorption conditions [29]. Two main types of intestinal perfusion models exist: unidirectional and circulating perfusion. Circulating perfusion typically employs higher flow rates, which may damage the intestinal mucosa and introduce errors during sampling or volume compensation [30]. In contrast, unidirectional perfusion uses lower flow rates (0.2 mL·min^−1^) and shorter durations (within 2 h), minimizing mucosal injury and better mimicking the physiological environment of orally administered drugs [31]. Therefore, the rat unidirectional intestinal perfusion model was adopted in this study. SPIP results showed that both SIN and SIN-LCNPs exhibited significantly higher Ka and Papp values in the small intestine than in the colon, indicating improved absorption in the small intestinal segment. Compared with SIN, SIN-LCNPs demonstrated a 1.36-fold increase in Ka and a 1.92-fold increase in Papp in the small intestine and a 2.22-fold and 2.67-fold increase in Ka and Papp, respectively, in the colon. These results indicate that LCNPs effectively enhance SIN permeability and absorption, thereby improving its oral bioavailability. The observed enhancement in small intestinal absorption can be attributed to both intestinal morphology and nanoparticle characteristics. Structurally, the small intestinal mucosa, densely covered with villi, provides a large surface area that promotes retention of amorphous nanocarriers, prolonging residence time and facilitating absorption [32]. Moreover, Peyer’s patches contain M cells capable of recognizing and internalizing nanoparticles, further promoting SIN-LCNP uptake [33,34,35,36]. In terms of formulation properties, the nanoscale particle size of SIN-LCNPs enhances drug dissolution, while the lipid-based matrix facilitates transmembrane transport. Within the intestinal mucus layer, SIN-LCNPs may form micellar or gel-like structures that reduce mucosal clearance and extend epithelial contact time [37]. Collectively, these structural and formulation-related features synergistically enhance intestinal absorption. The double-continuous mesh architecture and lipidic characteristics of LCNPs reduce adhesion to the mucus layer and improve mucosal penetration. Their internal cavities protect the encapsulated drug from gastric acid and digestive enzymes while promoting chylomicron association or M cell–mediated lymphatic transport, partially bypassing hepatic first-pass metabolism. This integrated mechanism—comprising mucosal penetration, drug protection, and lymphatic transport—jointly contributes to the improved intestinal absorption of SIN. From a translational standpoint, orally administered SIN-LCNPs may be suitable for long-term RA therapy and could be extended to other hydrophobic anti-inflammatory agents. The preparation process is compatible with continuous industrial-scale production, employing pharmaceutical-grade excipients and cost-controlled parameters. Optimized formulation and processing conditions meet quantifiable quality standards, demonstrating translational feasibility and scalability.

## 4. Materials and Methods

### 4.1. Materials

All reagents, including Sinomenine (98%, Chengdu Chenye Biotechnology Co., Ltd., Chengdu, China), glycerol monolaurate, Poloxamer 407, and phytolactone (Shanghai Yuanye Biotechnology Co., Ltd., Shanghai, China), conform to the standards for analytical grade purity and quality. SPF-grade Sprague–Dawley (SD) male rats, weighing 200 ± 20 g, were obtained from Liaoning Changsheng Biotechnology Co., Ltd., Shenyang, China (License No.: SCXK(Liao)2020-0001). The animal experiment protocol was approved by the Animal Ethics Committee of Changchun University of Chinese Medicine (Approval No.: 2024795), in accordance with institutional guidelines.

### 4.2. Excipient Screening

Lipid cubic liquid crystals consist of amphiphilic lipids, stabilizers, and high-purity water. According to interfacial stacking theory, amphiphilic lipids generally require a carbon chain length of at least 20 atoms. Currently, commonly used amphiphilic lipids for preparing cubic liquid crystals include GMO, PT, MGLA, and OEA [38]. Stabilizers improve the stability of lipid cubic liquid crystal drug delivery systems by preventing nanoparticle sedimentation or aggregation. Predominant stabilizers include surfactants such as P407, PVA, VEA, and CTAB [39].

MD simulations were employed to calculate δ of SIN and various excipients in order to evaluate their molecular compatibility. δ, a physical constant reflecting polymer miscibility, is defined as the square root of CED, as expressed in Equation (1) [40]. Generally, a difference in δ values (Δδ) below 7.5 indicates good miscibility between components [41,42].

(1) $$\delta =\sqrt{CED}=\sqrt{E_{coh}/V}$$

**Figure 8:**
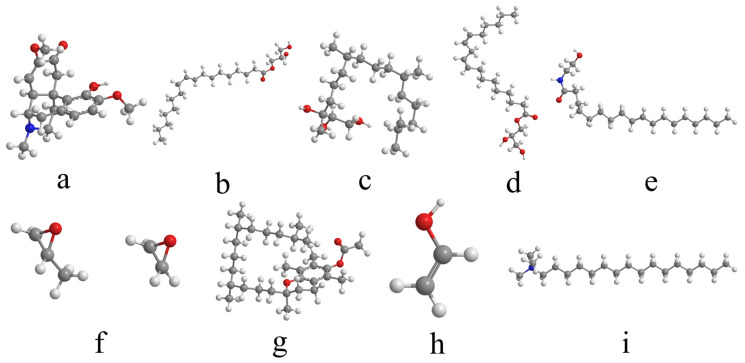
Three-dimensional molecular structure diagrams of excipient screening. (**a**) SIN; (**b**) GMO; (**c**) PT; (**d**) MGLA; (**e**) OEA; (**f**) P407; (**g**) VEA; (**h**) PVA; (**i**) CTAB. In all structures, gray represents carbon atoms, white represents hydrogen atoms, red represents oxygen atoms, and blue represents nitrogen atoms.

**Figure 9:**
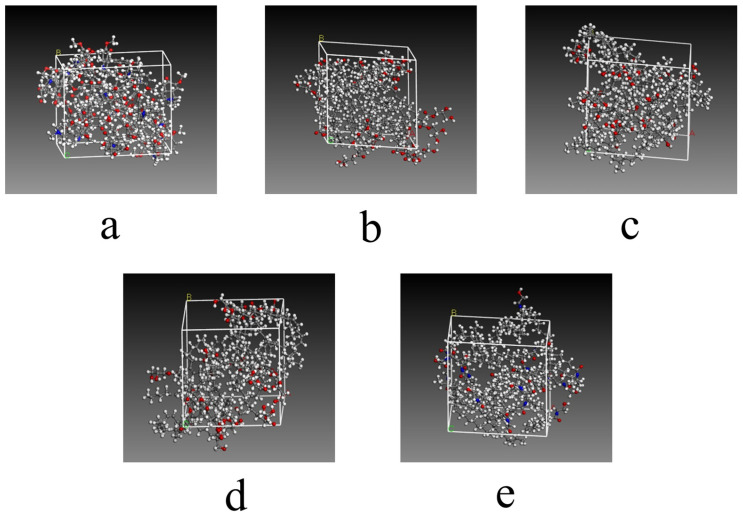
The optimized cell diagrams of excipient screening. (**a**) SIN; (**b**) GMO: The optimized unit cell structure of glycerol monooleate, showing carbon (gray), hydrogen (white), and oxygen (red) atoms forming typical ester and hydroxyl groups of the monoacylglycerol molecule, arranged in a stable configuration; (**c**) PT: The optimized unit cell structure of phytantriol, illustrating the branched alkyl chains (gray carbon and white hydrogen atoms) and trihydroxy functional groups (red oxygen atoms) that reflect the spatial conformation of this polyol excipient; (**d**) MGLA; (**e**) OEA. In all structures, gray represents carbon atoms, white represents hydrogen atoms, red represents oxygen atoms, and blue represents nitrogen atoms.

**Figure 10:**
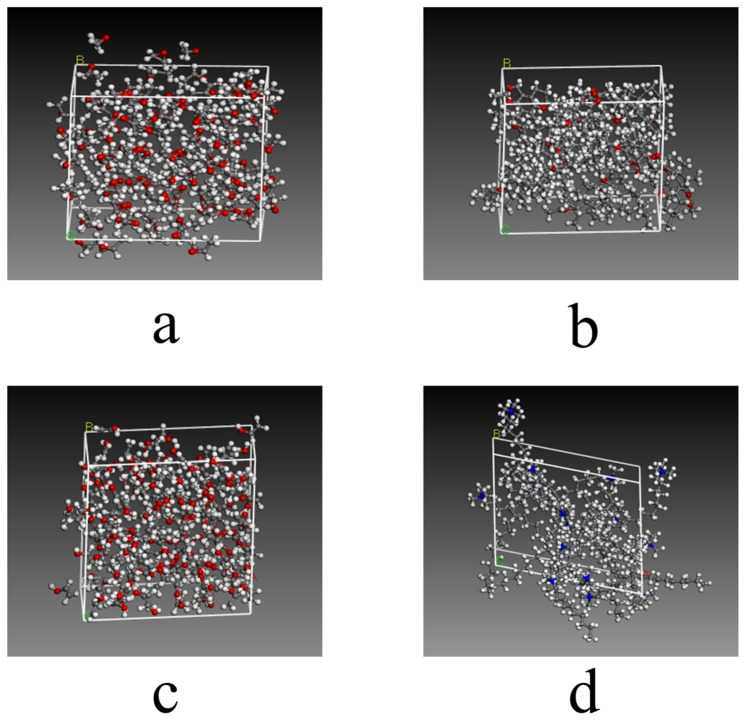
The optimized cell diagrams of excipient screening. (**a**) P407; (**b**) VEA: The optimized unit cell structure of vinyl ethyl ether copolymer, where red triangles indicate the ether functional groups (comprising red oxygen and gray carbon atoms) distributed along the polymer chain, reflecting the structural characteristics of the copolymer; (**c**) PVA: The optimized unit cell structure of polyvinyl alcohol, in which hydroxyl groups (red oxygen atoms) and alkyl backbones (gray carbon atoms) form a dense network; the red triangles mark hydrogen-bonded hydroxyl clusters within the system; (**d**) CTAB. In all structures, gray represents carbon atoms, white represents hydrogen atoms, red represents oxygen atoms, and blue represents nitrogen atoms.

**Figure 11:**
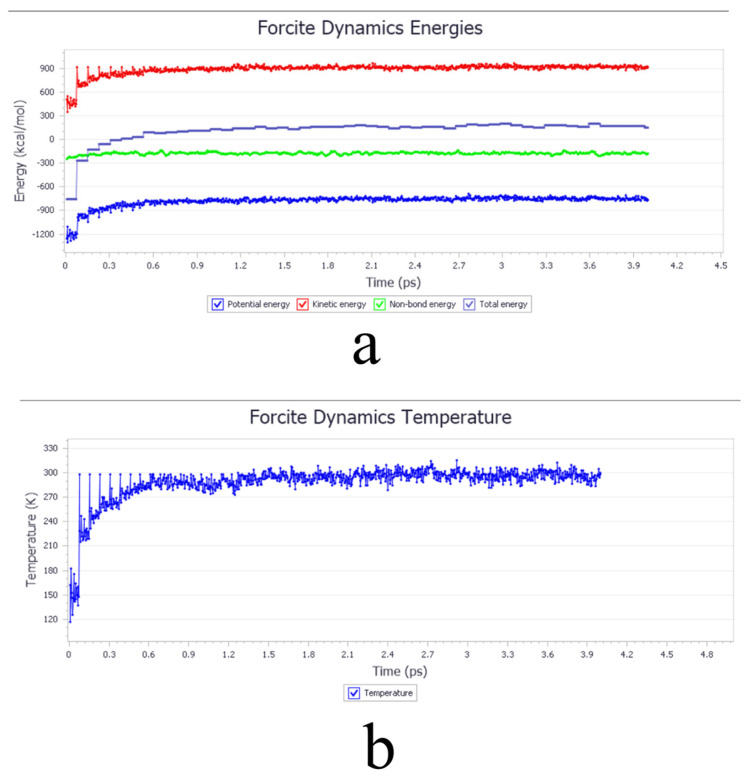
Excipient screening. (**a**) Energy versus time equilibrium curve. (**b**) Temperature versus time equilibrium curve.

The initial molecular structures were first obtained. The 3D structures of SIN, PT, MGLA, OEA, P407, PVA, and VEA were retrieved from the PubChem database, while those of GMO and CTAB were constructed using GaussianView 5.09. The 3D molecular structure diagrams are shown in Figure 8. Bond lengths and angles were manually adjusted to generate reasonable initial conformations and avoid steric conflicts. Each molecular structure was then optimized using the Forcite module with the COMPASS force field. Atomic charges were assigned automatically by the module’s built-in charge equilibration algorithm under ultra-fine precision. A Smart optimization with an initial step size of 0.01 Å was first applied to rapidly remove large internal stresses and obtain preliminary conformations with appropriate bond lengths and angles. Subsequently, the Conjugate Gradient method was executed for 500 iterations to minimize potential energy to a local minimum, yielding optimized structures with stable energy values and RMS gradients <0.001 kcal·mol^−1^·Å^−1^. A final Quasi-Newton optimization was performed for 2000 iterations using an energy convergence criterion of <1 × 10^−5^ kcal·mol^−1^, resulting in the lowest-energy conformations after four optimization cycles. Amorphous unit cells were then generated and optimized using the Amorphous Cell module. Cubic unit cells were constructed containing a single type of optimized molecule, and the same optimization parameters were applied as in the molecular-level optimization. The optimized cell diagrams are shown in Figure 9 and Figure 10. After four optimization cycles, the initially low-density, high-energy cells were transformed into stable, high-density unit cells. MD simulations were subsequently conducted in the NVT ensemble at 298 K using an Andersen thermostat, a 0.25 fs time step, and a total of 16,000 steps (4 ps simulation time). The COMPASS force field, charge assignments, and precision settings were consistent with previous stages. The non-bonded interaction cut-off was set to 12.5 Å, and long-range electrostatic interactions were computed using Ewald summation. As shown in Figure 11, the system energy reached stability with a potential energy standard deviation below 5% at 0.6 ps, and the temperature fluctuated within ±2 K of the 298 K setpoint by 1.5 ps, indicating that molecular equilibrium was achieved early. After equilibration, CED and δ were calculated using the Cohesive Energy Density task in Forcite. Each system was simulated three times under identical conditions to obtain averaged δ values and ensure reproducibility. All MD simulations were performed on a server equipped with a 16-core, 32-thread CPU running the Linux operating system. The server provides sufficient computational performance for equilibrium and production simulations, ensuring numerical stability and reproducibility of the results. The computational results were subsequently validated by experimental excipient screening, confirming the reliability of the simulation approach.

### 4.3. Optimization of SIN-LCNP Formulation and Process

Initial screening of relevant variables was carried out through single-factor experiments. The formulation parameters examined included the excipient ratio, moisture content, drug content, and excipient quantity, while the processing variables comprised stirring time, homogenization pressure, and number of homogenizations. Based on the results of these preliminary tests, a systematic optimization was performed using the Box–Behnken response surface methodology to explore the interactions among the key formulation factors. Three variables—excipient ratio (A), moisture content (B), and drug content (C)—were selected for detailed investigation. A three-factor, three-level experimental design was developed, with the responses being EE% (Y_1_) and particle size (Y_2_). This approach aimed to identify the optimal combination of formulation parameters to maximize EE% and minimize particle size for enhanced nanocarrier performance.

### 4.4. Preparation of SIN-LCNPs

Specified amounts of GMO, P407, and SIN were accurately weighed. SIN was ultrasonically dissolved in absolute ethanol until completely solubilized. GMO was placed in a beaker and melted in a water bath at 60 °C. The molten GMO was then mixed with the SIN ethanol solution under magnetic stirring at 60 °C to obtain Phase A. Separately, P407 was dissolved in ultrapure water under magnetic stirring at 70 °C until a clear solution was formed, constituting Phase B. Phase A was gradually added to Phase B under continuous stirring at 60 °C. After stirring for a designated period, high-pressure homogenization was immediately performed to obtain the SIN-LCNPs.

### 4.5. Method for Determining EE%

Accurate determination of EE% requires effective separation of nanoparticles from unencapsulated drug molecules to ensure analytical reliability. In this study, ultrafiltration centrifugation was employed based on molecular weight differences. Specifically, 1 mL of the SIN-LCNPs suspension was placed in a regenerated cellulose (RC) ultrafiltration membrane (0.22 μm pore size, 8000–12,000 Da molecular weight cut-off) and centrifuged at 12,000 rpm for 20 min at 4 °C. The filtrate was collected, and the amount of free SIN ($m_{free}$) was quantified using high-performance liquid chromatography (HPLC). Separately, 1 mL of the SIN-LCNPs suspension was diluted to volume with methanol to obtain a standard solution for determining the total SIN content ($m_{total}$) by HPLC. The EE% was calculated using Equation (2). HPLC analysis was performed under the following conditions: Agilent 5 TC-C18 column (4.6 mm × 250 mm, 5 μm); mobile phase consisting of methanol and 0.05 M sodium dihydrogen phosphate buffer (20:80, *v*/*v*); flow rate of 1.0 mL min^−1^; detection wavelength of 262 nm; column temperature of 30 °C; and injection volume of 10 μL.

(2) $$EE\%=(1-\frac{m_{free}}{m_{total}})\times 100\%$$

### 4.6. Characterization of SIN-LCNPs

#### 4.6.1. Appearance, Particle Size, PDI, and Zeta Potential Analysis

The particle size distribution of SIN-LCNPs was measured using a nanolaser particle size analyzer (Mastersizer 3000, Malvern Panalytical, Worcestershire, UK). The prepared SIN-LCNPs were stored in transparent vials at room temperature (RT, 20–25 °C) and equilibrated for 24 h prior to analysis. The morphological appearance was examined via macroscopic observation and recorded through photographs. An aliquot of the SIN-LCNPs was diluted 30-fold with ultrapure water to prepare the sample for particle size analysis. A 1 mL portion of the diluted sample was measured for particle size, PDI, and zeta potential using a nanoparticle size analyzer at room temperature. Each measurement was performed in triplicate to ensure reproducibility. The results are expressed as mean ± standard deviation.

#### 4.6.2. FT-IR Analysis

FT-IR spectra were recorded on a Nicolet iS50 spectrometer (Thermo Scientific, Waltham, MA, USA). A small amount of each sample (SIN, GMO, physical mixture, SIN-LCNPs) was thoroughly ground with an appropriate amount of dried potassium bromide, typically in a mass ratio of 1:100. The mixture was then pressed into a uniform, thin pellet using a hydraulic press. FT-IR spectra were recorded using a spectrometer over the wavenumber range of 4000–400 cm^−1^, following background subtraction. The spectral resolution was set between 4 and 8 cm^−1^. All measurements were performed at room temperature (RT, 20–25 °C). Each spectrum was obtained by averaging 32 scans to improve signal-to-noise ratio.

#### 4.6.3. TEM Morphological Analysis

TEM was performed using a Talos F200X G2 microscope (Thermo Scientific, Waltham, MA, USA) to observe nanoparticle morphology. A small volume (approximately 5 μL) of the nanoemulsion was carefully deposited onto a carbon-coated copper grid. The sample was allowed to stand for 30 min at room temperature to facilitate adhesion. Excess liquid was gently blotted off using filter paper to leave a thin, uniform film. The grid was then negatively stained with 2.0% phosphotungstic acid solution, diluted appropriately, and incubated for approximately 2 min to enhance contrast. After staining, the grid was air-dried at room temperature and then examined under a transmission electron microscope at an accelerating voltage of 80 kV. Images were captured at suitable magnifications to analyze the morphology and size distribution of the SIN-LCNPs.

#### 4.6.4. PLM Analysis

PLM analysis was conducted using a DM2700P microscope (Leica Microsystems, Wetzlar, Germany) to examine the liquid crystalline structure. Liquid crystals with different molecular arrangements display characteristic optical behaviors under PLM. Cubic liquid crystals are generally optically isotropic, appearing highly transparent with no birefringence, and thus produce a uniformly dark field under PLM, characteristic of an isotropic phase [43]. A small volume (approximately 5 μL) of SIN-LCNPs dispersion was evenly spread onto a clean glass slide. The sample was then covered with a coverslip, carefully pressed to eliminate air bubbles, ensuring a flat and uniform sample surface. The prepared slide was observed under a polarized light microscope equipped with crossed polarizers at 40× magnification. The optical properties, such as birefringence or isotropic nature, were examined to identify the liquid crystalline phase.

### 4.7. Pharmacodynamic Study of SIN-LCNPs in AA Rats

#### 4.7.1. Establishment of the Rat AA Model

Healthy Sprague–Dawley rats (male, 8–10 weeks old, weighing 200–250 g) were housed under standard conditions (22 °C, 50–60% humidity, 12 h light/dark cycle). After a one-week acclimatization period with free access to food and water, arthritis was induced by intradermal injection of complete Freund’s adjuvant (CFA; containing 10 mg/mL of oil phase) into the right hind toe. Specifically, 0.1 mL of CFA solution was injected into the subdermal tissue of the toe to establish the AA model. The control group received an equal volume of sterile physiological saline via the same injection site on Day 0. All procedures were conducted in accordance with institutional animal care guidelines and were approved by the relevant ethics committee.

#### 4.7.2. Grouping and Administration in Rats

On Day 0, male Sprague–Dawley rats were injected intradermally with 0.1 mL of CFA into the right hind paw to induce arthritis. Following one week of adaptation, the rats were monitored, and by Day 15, signs of immune and inflammatory responses appeared in the contralateral paw. An arthritis index score of ≥4 was considered indicative of successful model induction [44]. Rats with successful induction were randomly divided into four groups (*n* = 6 per group): AA model, Dex, SIN, and SIN-LCNPs. Treatments were initiated on Day 15, with all groups receiving oral gavage once daily for 21 consecutive days. The doses were calculated based on body surface area normalization, using 216 mg/day of SIN as the human equivalent dose, which corresponds to approximately 19.44 mg/kg/day in rats [45]. The Dex group received 1.08 mg/kg/day. The NC group, with 6 animals, and the AA group received an equivalent volume of physiological saline via oral gavage daily. All procedures adhered to institutional animal care guidelines and were approved by the relevant ethics committee.

#### 4.7.3. Measurement of Paw Edema

Paw swelling was measured before model induction (Day 0) and every 3 days thereafter (Days 3, 6, 9, 12, 15, 18, 21, 24, 27, 30, 33, and 36). Using a vernier caliper, the diameter of the left and right paw joints was recorded. The caliper jaws were gently positioned around the mid-region of each paw or at the joint level, ensuring contact without applying pressure that could distort measurements. All measurements were performed with the rats restrained in a consistent position, and each measurement was repeated three times to obtain an average value. Experiments were conducted in a stable environment at room temperature.

#### 4.7.4. Arthritis Index Assessment

Following the confirmation of successful arthritis induction, the arthritis index was evaluated every 3 days (Days 3, 6, 9, 12, 15, 18, 21, 24, 27, 30, 33, and 36). The severity of joint inflammation was scored based on visual assessment of erythema, swelling, and induration of each paw [46], according to the following criteria:

0 points: No signs of inflammation.

1 point: Few erythematous lesions with mild edema involving the ankle joint.

2 points: Moderate erythema and edema involving the ankle joint and metacarpal bones.

3 points: Numerous erythematous lesions, severe edema encompassing the metatarsals or phalanges.

4 points: Severe joint deformity, involving the entire paw and impairing normal function.

The scores from all four limbs were summed to obtain each rat’s total arthritis index, with a maximum possible score of 16. Higher scores indicated more severe arthritis symptoms. All assessments were performed by blinded evaluators trained to maintain consistency.

#### 4.7.5. Serum Inflammatory Cytokine Assay

Following the final administration of the treatment, all rats were fasted for 12 h with free access to water. Rats were anesthetized via intraperitoneal injection of 20% urethane solution at a dose of 5 mL/kg. Blood was collected from the abdominal aorta using sterile syringes and allowed to clot at room temperature for 30 min. The blood samples were then centrifuged at 3500 rpm for 10 min, and the serum was carefully aspirated, aliquoted into sterile microtubes, and stored at −80 °C until further analysis. Serum concentrations of TNF-α, IL-1β and IL-6 were quantified using commercially available ELISA kits, following the manufacturer’s protocols. All assays were performed in duplicate, and the absorbance was measured at the specified wavelength (typically 450 nm) using a microplate spectrophotometer. Calibration curves were prepared with standard samples to ensure quantification accuracy.

#### 4.7.6. Determination of Rat Spleen Index and Thymus Index

On Day 37, rats were euthanized after blood collection. The spleen and thymus were carefully dissected from each rat, blotted dry, and weighed using an electronic analytical balance. The spleen index and thymus index were calculated with the following Formula (3):

Spleen Index (%) = (Spleen weight/Body weight) × 100 Thymus Index (%) = (Thymus weight/Body weight) × 100(3)

All weights were measured at room temperature, and indices were used to evaluate immune organ atrophy or hypertrophy in response to treatment.

#### 4.7.7. HE Staining for Pathological Observation

The right hind ankle joints were carefully dissected and fixed in 4% paraformaldehyde at 4 °C for 48 h. Following fixation, the tissues were dehydrated through ascending concentrations of ethanol (70%, 80%, 95%, 100%), cleared in xylene, and embedded in paraffin wax. Serial sections of approximately 4 μm thickness were sliced using a rotary microtome, mounted on glass slides, baked at 60 °C for 1 h, and stored at room temperature until staining. For HE staining, sections were deparaffinized by immersing in xylene, rehydrated through a decreasing ethanol series, and washed in water. Hematoxylin staining was performed for 5 min, followed by rinsing and differentiation with hydrochloric acid alcohol solution (1% HCl in 70% ethanol) until nuclei were distinctly stained. The sections were then blued in ammonia methyl green or Masson’s blue for 30 s, rinsed in water, and counterstained with eosin for 2 min. After dehydration through graded ethanol and clearing with xylene, slides were mounted with neutral resin and examined under a light microscope (magnification 100×). Images were captured and saved digitally for subsequent analysis.

### 4.8. SPIP Study of SIN-LCNPs

#### 4.8.1. Preparation of Perfusion Solutions

##### Preparation of Artificial Intestinal Fluid (Krebs–Ringer’s Solution, K-R Solution)

An appropriate amount of sodium chloride (7.8 g), potassium chloride (0.35 g), sodium bicarbonate (1.37 g), sodium dihydrogen phosphate (0.32 g), calcium chloride (0.37 g), magnesium chloride (0.02 g), and glucose (1.4 g) were accurately weighed and transferred into a clean volumetric flask. Distilled water was added to dissolve the constituents thoroughly, assisted by ultrasonication for 30 min. The solution was then adjusted to pH 7.4 using a calibrated pH meter before being diluted to a final volume of 1000 mL with distilled water. The prepared K-R solution was stored at room temperature and used immediately for intestinal perfusion experiments.

##### Preparation of the SIN Active Pharmaceutical Ingredient (API) Perfusion Solution

First, 30 mg of SIN was accurately weighed and transferred into a 200 mL volumetric flask. The API was dissolved initially in a small volume of K-R solution with gentle agitation. The solution was then diluted to a final volume of 200 mL with additional K-R solution to obtain a concentration of 150 μg/mL. Afterwards, 0.3% sodium carboxymethyl cellulose suspension was added as a dispersing agent. The mixture was sonicated for 30 min using an ultrasonic processor to achieve a uniform dispersion. The prepared solution was used immediately or stored at 4 °C for no longer than 24 h to prevent degradation.

##### Preparation of SIN-LCNPs Perfusion Solution

First, 19.365 mL of SIN-LCNPs were accurately weighed and transferred into a 200 mL volumetric flask. K-R solution was added to the flask and diluted to a final volume of 200 mL. The mixture was sonicated for 30 min using an ultrasonic processor to achieve uniform dispersion. The resulting SIN-LCNPs perfusion solution had a mass concentration of 150 μg/mL, which should be used immediately for in vivo studies or stored at 4 °C for no more than 24 h.

#### 4.8.2. Perfusate Collection and Analysis

Prior to the experiment, the perfusion tubing was flushed with the drug solution to ensure that the drug concentration at the outlet was equilibrated with that in the perfusion solution. Twelve rats, fasted for 24 h with unrestricted access to water, were randomly divided into two groups: the SIN group and the SIN-LCNP group, with six rats per group. Anesthesia was induced by intraperitoneal injection of a 20% urethane solution. Once the rats were fully anesthetized, a small midline abdominal incision was made to minimize trauma. Target intestinal segments (either small intestine or colon) were carefully dissected, and a 10 cm length was measured for the perfusion experiment. Small incisions were made at both ends of the dissected segment to allow for perfusion tube insertion. The segment was first flushed with 37 °C physiological saline to remove intestinal contents. The inflow and outflow perfusion tubes were connected to the segment with a 1 cm length reserved at each tube end for fixation. The segment was equilibrated for 15 min using K-R solution at 37 °C and a perfusion rate of 1 mL/min, followed by a 40 min equilibration using the test perfusion solution at 37 °C and a flow rate of 0.2 mL/min. Perfusion was then initiated using pre-weighed Epitron tubes containing the test perfusion solution at a flow rate of 0.2 mL/min. Effluent samples were collected in pre-weighed receiving bottles at 15 min intervals for a total of six collections. The incision site was covered with moist gauze to retain moisture, and infrared lamps were used to maintain the temperature of the wound. After the experiment, the rats were euthanized. The intestinal segments were excised, and their length and internal radius were measured using a surgical thread attachment method. The weights of the vials containing the perfusion solution were recorded, and the amounts of infused and effluent drug solutions were calculated. Effluent samples were centrifuged at 12,000 rpm for 10 min, and the supernatant was collected, diluted with methanol, and analyzed by HPLC to determine SIN content. The inflow and outflow volumes of the perfusion fluid were corrected using the gravimetric method. Ka and Papp were then calculated according to the following Formula (4) [47]:

(4) $$\mathrm{Ka}\;=\;(1-C_{out}Q_{\mathrm{out}}/C_{in}Q_{in})Q/\pi r^{2}l\;\mathrm{Papp}\;=\;-Q\ln(C_{out}Q_{\mathrm{out}}/C_{in}Q_{in})/2\pi \mathrm{rl}$$

C_in_ and C_out_ represent the concentrations of SIN (μg/mL) detected at the inlet and outlet of the perfusion fluid, respectively. Q_in_ and Q_out_ refer to the volumes of perfusion fluid (mL) at the intestinal inlet and outlet, respectively. r is the cross-sectional radius (cm) of the perfused intestinal segment. l denotes the length (cm) of the perfused intestinal segment. Q represents the flow rate (mL/min) of the perfusion solution, and t indicates the perfusion duration for each 15 min interval.

### 4.9. Statistical Analysis

Data are expressed as mean ± standard error of the mean (SEM) for normally distributed data or as median with interquartile range (IQR) for non-normal data. Normality was assessed using the Shapiro–Wilk test. Comparisons between two groups were performed using unpaired Student’s *t*-test if data were normally distributed, or the Mann–Whitney U test if not. For multiple group comparisons, one-way ANOVA followed by Tukey’s post hoc test was used. Correlations between variables were analyzed with Pearson’s correlation coefficient for normally distributed data or Spearman’s rank correlation for non-normal data. *p*-values less than 0.05 were considered statistically significant, with * *p* < 0.05, ** *p* < 0.01, and *** *p* < 0.001 indicating increasing levels of significance. All analyses were conducted using GraphPad Prism version 9.5.

## 5. Conclusions

This study innovatively integrates MD simulations with traditional Chinese medicine formulation techniques, effectively overcoming the limitations of conventional trial-and-error screening approaches. Through this combined strategy, the optimal formulation and preparation process for SIN-LCNPs were efficiently identified. The resulting nanoparticles exhibited nanoscale dimensions, uniform dispersion, and high encapsulation efficiency. The results indicate that SIN-LCNPs significantly enhance targeted drug delivery to inflamed joints, alleviating arthritis symptoms and inhibiting disease progression in rats while demonstrating superior safety and biocompatibility compared with Dex. Furthermore, SIN-LCNPs improved drug absorption and permeability in both the small intestine and colon, highlighting their advantages for oral delivery. Overall, LCNPs demonstrate considerable potential as oral nanocarrier systems. This study not only provides an effective strategy for optimizing SIN formulations but also offers valuable insights for developing other poorly water-soluble drugs. Moreover, the MD simulation-guided approach to drug carrier design establishes a methodological framework for advancing precision research in pharmaceutical nanotechnology.

## Figures and Tables

**Table 1:** δ values of SIN and candidate excipients (*n* = 3).

Substance Name	δ (J/cm^3^)^1/2^	Δδ (J/cm^3^)^1/2^	RSD%
SIN	22.122	-	0.58
GMO	21.720	0.402	0.54
PT	19.730	2.392	1.69
linoleic acid monoglyceride (MGLA)	30.652	8.470	0.37
oleyl ethanolamine (OEA)	13.934	8.188	0.80
P407	24.337	2.215	0.54
polyvinyl alcohol (PVA)	29.614	7.592	0.14
vitamin E acetate (VEA)	14.084	8.038	1.36
hexadecyl trimethyl ammonium bromide (CTAB)	29.682	7.560	0.67

**Table 2:** Determination Results of SIN-LCNPs with Different Lipid Materials.

Lipid Materials	Appearance	Particle Size (nm)	EE%
GMO	Pale yellow milky liquid	162.8	86.6
PT	Pale yellow milky liquid	289.9	51.1

**Table 3:** Study design and results.

Test Number	A Excipient Ratio	B Moisture Content	C Drug Content	Y_1_ EE/%	Y_2_ Particle Size/nm
1	9:1	10:1	1.5%	82.1	176.8
2	15:1	10:1	1.5%	81.3	167.4
3	9:1	14:1	1.5%	74.5	154.9
4	15:1	14:1	1.5%	73.1	176.8
5	9:1	12:1	1%	74.6	145
6	15:1	12:1	1%	76.7	169.4
7	9:1	12:1	2%	77.4	167.4
8	15:1	12:1	2%	74.9	160.6
9	12:1	10:1	1%	89.0	159.0
10	12:1	14:1	1%	78.6	147.5
11	12:1	10:1	2%	86.6	153.6
12	12:1	14:1	2%	88.4	155.9
13	12:1	12:1	1.5%	92.9	149.8
14	12:1	12:1	1.5%	93.7	149.8
15	12:1	12:1	1.5%	89.8	149.8
16	12:1	12:1	1.5%	93.5	151.4
17	12:1	12:1	1.5%	93.2	147.5

**Table 4:** Y_1_ ANOVA results.

Source of Variance	Sum of Squares of Mean Deviation	Free Degree	Mean Square	F-Value	*p*-Value
model	924.29	9	102.7	32.22	<0.0001
A	0.8450	1	0.8450	0.2651	0.6225
B	74.42	1	74.42	23.35	0.0019
C	8.82	1	8.82	2.77	0.1402
AB	0.0900	1	0.0900	0.0282	0.8713
AC	5.29	1	5.29	1.66	0.2386
BC	37.21	1	37.21	11.67	0.0112
A2	638.05	1	638.05	200.17	<0.0001
B2	27.59	1	27.59	8.66	0.0216
C2	81.89	1	81.89	25.69	0.0014
residual	22.31	7	3.19	-	-
Lost item	12.01	3	4.00	1.55	0.3319
Pure deviation	10.31	4	2.58	-	-
Total deviation	946.60	16	-	-	-

**Table 5:** Y_2_ ANOVA results.

Source of Variance	Sum of Squares of Mean Deviation	Free Degree	Mean Square	F-Value	*p*-Value
model	1662.39	9	184.71	48.96	<0.0001
A	113.25	1	113.25	30.02	0.0009
B	58.86	1	58.86	15.60	0.0055
C	34.44	1	34.44	9.13	0.0193
AB	244.92	1	244.92	64.92	<0.0001
AC	243.36	1	243.36	64.50	<0.0001
BC	47.61	1	47.61	12.62	0.0093
A2	706.93	1	706.93	187.38	<0.0001
B2	170.18	1	170.18	45.11	0.0003
C2	17.14	1	17.14	4.54	0.0705
residual	26.41	7	3.77	-	-
Lost item	18.66	3	6.22	3.21	0.1448
Pure deviation	7.75	4	1.94	-	-
Total deviation	1688.80	16	-	-	-

## Data Availability

The original contributions presented in this study are included in the article. Further inquiries can be directed to the corresponding authors.
